# Mechanical and Shape Memory Properties of Additively Manufactured Polyurethane (PU)/Halloysite Nanotube (HNT) Nanocomposites

**DOI:** 10.3390/nano14161373

**Published:** 2024-08-22

**Authors:** Wendy Triadji Nugroho, Yu Dong, Alokesh Pramanik

**Affiliations:** 1 School of Civil and Mechanical Engineering, Curtin University, GPO Box U1987, Perth, WA 6845, Australia; wendy@polije.ac.id (W.T.N.); alokesh.pramanik@curtin.edu.au (A.P.); 2 Engineering Department, Politeknik Negeri Jember, GPO Box 164, Jember 68101, Indonesia

**Keywords:** polyurethane (PU), halloysite nanotube (HNT), additive manufacturing, mechanical property, shape memory property

## Abstract

This paper investigates the impact of halloysite nanotube (HNT) content on mechanical and shape memory properties of additively manufactured polyurethane (PU)/HNT nanocomposites. The inclusion of 8 wt% HNTs increases their tensile strength by 30.4% when compared with that of virgin PU at 44.75 MPa. Furthermore, consistently significant increases in tensile modulus, compressive strength and modulus, as well as specific energy absorption are also manifested by 47.2%, 34.0%, 125% and 72.7% relative to neat PU at 2.29 GPa, 3.88 MPa, 0.28 GPa and 0.44 kJ/kg respectively. However, increasing HNT content reduces lateral strain due to the restricted mobility of polymeric chains, leading to a decrease in negative Poisson’s ratio (NPR). As such, shape recovery ratio and time of PU/HNT nanocomposites are reduced by 9 and 45% with the inclusion of 10 wt% HNTs despite an increasing shape fixity ratio up to 12% relative to those of neat PU.

## 1. Introduction

**Figure 1:**
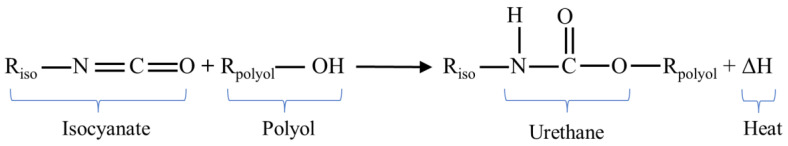
Urethane formation from isocyanate and polyol [12].

PU is made through a chemical reaction that involves isocyanate functional groups (NCO) and hydroxyl (OH) groups of polyols. The forming reaction of PU is exothermic and releases an amount of energy via either heat or light, as described in Figure 1, where *R_iso_* and *R_polyol_* denote isocyanate monomers and polyol components respectively. Generally speaking, the chemical structure of PU consists of two segment types, namely soft and hard segments, which are constructed in an alternating mode. Soft segments (i.e., oligodiol) with low glass transition temperature *T_g_* create continuous matrices possessing flexibility characteristics at low temperatures. Meanwhile, hard segments (with high *T_g_*) can perform self-assembly in particular areas via crosslinking [1]. These areas can be reinforcements in continuous matrices with low *T_g_* and improve material performance, including solvent resistance, mechanical strength and thermal properties [1]. Thermoplastics and thermosets in PU family exhibit shape memory effect (SME), which is also referred to as shape memory behaviour of PU. The primary distinction between ordinary PU and shape memory PU is within the range of their *T_g_*. PU commonly possesses a larger range of *T_g_* that can maintain its deformed shape after being heated above *T_g_* when subjected to a mechanical load and then cooled below *T_g_*. By implementing an external stimulus, such as heating above *T_g_*, PU finally regains its initial shape. Therefore, this material has been widely implemented in widespread applications, which are not limited to scaffolds [2], biomedical devices [3], finger orthosis [4], biomimicked skeletal muscle actuators [5], wearable devices [6], strain sensors [7], biomaterials [8], coatings [9], structural foams [10], as well as special generators made from stretchable organic elements [11].

Virgin PU has remarkable elongation up to 400% despite its low mechanical strength. The presence of nanofillers, such as multi-walled carbon nanotubes (MWCNTs), improves Young’s modulus, as well as tensile strength of PU. It was reported that incorporating 3 wt% MWCNTs in PU matrices increased Young’s modulus and tensile strength of corresponding composites by approximately 39% and 49% respectively [13]. Nevertheless, when MWCNT content was increased to 5 wt%, there was a decrease in the tensile strength of PU/MWCNT composites by 72.24%, but their Young’s modulus was still enhanced by 20.17% [11]. Montmorillonite (MMT), another type of popular nanoparticles, can improve the capacity of dye absorption and hydrophilicity, as well as antifouling properties of PU-based fibrous membranes. The inclusion of 20 wt% MMTs in PU matrices reduced the water contact angle of nanocomposite membranes by 57° compared to virgin fibrous PU at 117°. In addition, at this MMT content level, nanocomposite membranes also had remarkable water flux and oil rejection required by antifouling membranes, which are usually implemented in wastewater treatment [14]. HNT particles were employed by Maamoun et al. [15] to enhance the capability of sound absorption for PU. The inclusion of such nanofillers up to 1 wt% successfully improved the coefficient of sound absorption for nanocomposite samples. 

One of key mechanical properties investigated in this study is negative Poisson’s ratio (NPR). To determine NPR, a re-entrant (RE) structure was utilised mainly because of its auxetic behaviour. A RE structure possesses a unique construction consisting of a number of well-designed cells, allowing it to possess lightweight and high-strength material characteristics due to its high stiffness/strength-to-mass ratio. Such a structure is also called auxetics especially with NPR, as indicated by its ability to reveal transverse expansion when subjected to uniaxial tension and transverse contraction under uniaxial compression [16]. Lakes [17] reported RE structures with extraordinary shear stiffness for the first time and embarked on the creation and development of various auxetic structures over many decades. Additionally, such great efforts have been made with respect to structure modelling and manufacturing, material characterisation, as well as modelling approaches of RE structures. In particular, finite element analysis (FEA), deemed one of the most effective modelling approaches, gains its popularity in predicting auxetic properties of RE structures, such as NPR and other key multifunctional properties, consisting of energy absorption, strain recovery and piezoresistance [18,19,20]. The successful progress of RE structures used in the fabrication od 3D objects enables to overcome critical issues with respect to complicated geometry and a lack of additive manufacturing methods. 

Additive manufacturing, also known as 3D printing, is classified as advanced manufacturing technology by means of adding materials successively to build designed parts with reference to 3D models created via computer-aided design (CAD) software such as SolidWorks 2023 and Fusion 360. Through this manufacturing method, material waste can be significantly reduced [21]. In addition, additive manufacturing is capable of creating 3D objects with complicated geometry and high porosity, as evidenced by RE structures with their auxetic characteristics. additive manufacturing has been implemented by General Electric (GE) Aviation to reduce fabrication costs to 75% in the assembling step for cast components, and thus saved USD 3 million per aircraft a year [22]. Furthermore, 3D printing has been used in many applications, especially for marine science, sports equipment, biomedical sciences, electronics, automobiles and food [12].

Even though additive manufacturing has many superiorities, it is unable to completely substitute conventional manufacturing technologies like extrusion and injection moulding, since it possesses typical disadvantages with respect to manufacturing time, surface quality and mechanical properties of 3D printed parts. Fused deposition modelling (FDM) is deemed the most popular additive manufacturing technique, especially based on plastics and polymer composites, with low fabrication cost [23]. FDM employs computer-aided design (CAD) in creating 3D models, and then their format is changed into Standard Tessellation Language (STL) in order to be processed by a 3D printer. Kokcu et al. [24] used FDM to fabricate nanocomposite scaffolds made from polylactic acid (PLA) and HNTs as the matrices and reinforcements respectively. The results indicated that the addition of 3 wt% HNTs significantly enhanced tensile, compressive and flexural strengths of 3D printed scaffolds by 124%, 145% and 41% accordingly. FDM was reported by Lv et al. [25] to successfully prepare flexible nanocomposites made from graphene-modified polyolefin elastomer (POE) for particular applications, such as shielding for electromagnetic interference and thermal management. The addition of 10.93 vol% graphene nanoplatelets could enhance the efficiency of electromagnetic shielding and thermal conductivity of 3D printed nanocomposites up to 35 dB and 4.3 W/m∙K (1600% greater than that of virgin POE). 

PU/HNT nanocomposites, additively manufactured by FDM, have also been mentioned in previous studies [26,27,28] as to the influence of HNTs on mechanical properties and cytotoxicity of 3D printed samples. It was found that tensile strength and elongation at break for nanocomposite samples were increased by 27% (from 26.04 to 33.12 MPa) and 50% (from 723 to 1085%) relative to those of virgin PU with the inclusion of 2 wt% HNTs. Material samples were prepared via melt mixing using a twin-screw extruder and injection moulding machine, as well as 3D printing with the aid of an FDM printer. The impact of HNT content on thermal stability and sound absorption of PU/HNT nanocomposites was thoroughly investigated by Mahunaki et al. [27]. It was revealed that the presence of 1 wt% HNTs enhanced *T_g_* of corresponding 3D printed nanocomposites because HNTs, as rigid nanofillers, reduced the movement of soft segments in molecular chains. Furthermore, at this HNT content, nanocomposite samples had better sound absorption than virgin PU, as indicated by the change from a mid-frequency to a high-frequency range. It was noteworthy that the samples were made using prepolymer and melt compounding methods. Another study conducted by Prasanthi et al. [28] implemented a dip-coating method. It was mentioned that the sorption capacity of 3D printed sponges made from PU, HNT particles and fluorinated graphene was in the range of 38–62 g/g with extraordinary recyclability when subjected to static and turbulent conditions, as well as remarkable corrosive and thermal stabilities.

Several studies [15,29,30,31] related to PU/HNT nanocomposites using conventional manufacturing methods have been widely conducted. However, comprehensive work regarding HNT inclusion on mechanical and shape memory properties of additively manufactured PU/HNT nanocomposites has rarely been investigated, which is a primary focus of this study. A common issue with nanocomposites is how to obtain material samples with an excellent dispersion of HNTs within PU matrices. Accordingly, solution casting was employed to overcome such a critical issue. It was then followed by an extrusion process to prepare nanocomposite filaments, which were used as feedstock materials for 3D printing to create particular nanocomposite structures. It aimed to evaluate mechanical and shape memory properties of PU/HNT nanocomposites for potential applications of biomedical and aerospace engineering.

## 2. Materials and Methods

### 2.1. Materials

**Figure 2:**
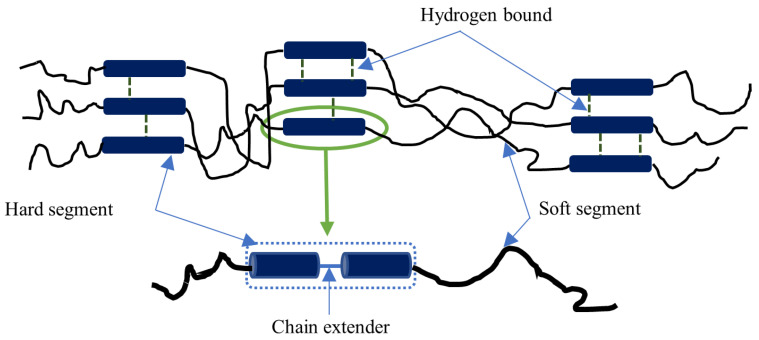
Chemical structure of PU [12].

This study used PU MM-4520 grade as the base polymer, which was fabricated by SMP Technologies Inc. [32], Tokyo, Japan. It has a *T_g_* of 45 °C and a melt viscosity of 3310 Pa∙s at 215 °C. The other important properties of PU MM-4520 grade are also mentioned in Table 1. HNT particles as the nanofillers were supplied by Imerys Ceramics [33], Matauri Bay, New Zealand. As listed in Table 2, such HNT nanoparticles possess essential properties, such as moisture content of 3%, specific gravity of 2.55, pH level of 3.5–4.5, surface area of 25 m^2^/g, linear shrinkage of 3.8% and modulus of rupture of 2.9 MPa. Meanwhile, dimethylformamide (DMF) was provided by ChemSupply, Gillman, SA, Australia, which was used as a chemical solvent to dissolve PU pellets. All materials were utilised without any modifications. Figure 2 displays PU chemical structure with hard and soft segments. Hard segments are constructed from diisocyanate and chain extender, while soft segments contain oligodiol. HNTs, as the additives in this study, are clay-based nanomaterials associated with kaolin family with unique hollow and tubular forms and high aspect ratios. Such a material has a chemical formula of Al_2_Si_2_O_5_(OH)_4_·nH_2_O. In general, well-dispersed HNTs possess a length of 100–2000 nm, as well as inner and outer diameters of 1–30 and 30–50 nm respectively [34].

### 2.2. Sample Preparation

HNT dispersion in PU matrices plays a crucial role in enhancing tensile and compressive properties of PU/HNT nanocomposites. This study involves three major material processing steps, namely drying process, dissolving process and mixing process. First of all, the moisture content of raw materials, including PU and HNTs, should be minimised using a vacuum oven to facilitate their separation in the mixing process. This is especially the case for HNT powders subjected to a drying process. Both PU pellets and HNT powders were dried in a vacuum oven at 80 °C for 4 and 8 h respectively. The solvent should be carefully selected to completely dissolve PU pellets. Furthermore, an appropriate mixing method needs to be applied in order to induce effective HNT dispersion within PU matrices. Accordingly, DMF was used as a chemical solvent, while heating, stirring and ultrasonication were deemed main mixing processes. 

HNT powders were dispersed in DMF at a weight ratio of 1:30 using an ultrasonication bath with a power intensity of 90%, a frequency of 25 kHz and a sonication time of 1 h. PU pellets and DMF were mixed at a weight ratio of 1:8. Prior to mixing, DMF was heated using a magnetic stirrer at a temperature of 300 °C with the rotor speed of 700 rpm. PU pellets were then continually added in small amounts to the beaker in order to avoid material sedimentation issues during the dissolution. Once all PU pellets were dissolved, HNT/DMF mixture was poured into PU/DMF solution under continuous stirring and heating processes. After 30 min, PU/HNT/DMF mixture was cast into a glass container and then heated in an oven at 100 °C for 24 h for DMF evaporation. Finally, prepared PU/HNT nanocomposite films were stored in a desiccator with silica gels before further material processing. It should be addressed that polymeric solutions were initially prepared to manufacture PU/HNT nanocomposite films.

Subsequently, these films were shredded and further chopped into small flakes (thickness: 0.45 ± 0.1 mm, width: 3.00 ± 0.1 mm and length: 4.30 ± 0.5 mm) with the aid of a paper shredder. Before the extrusion process, these flakes were dried in a vacuum oven at 80 °C for 4 h to reduce their moisture content. They were then fed into a filament extruder Filabot EX6, supplied by Filabot Company, Montpelier VT, USA, and underwent a melt mixing process to produce nanocomposite filaments used for subsequent 3D printing. This single-screw extruder has a screw diameter of 16 mm and an L/D ratio of 24. Four temperature zones of the extruder were set to 165, 165, 165 and 45 °C, and the screw speed was 50 rpm. Fabricated filaments were spooled and stored in airtight desiccator with silica gels.

An FDM printer Axiom 20 with APEX 1.7.4 slicer software, provided by Airwolf 3D, Costa Mesa, CA, USA, was used to process nanocomposite filaments. Detailed information regarding different manufacturing stages of 3D printed PU/HNT nanocomposites is available in our previous study [35].

The effect of HNT content and 3D printing parameters on tensile strength of PU/HNT nanocomposites were investigated using an *L*_18_ Taguchi orthogonal array (OA) listed in Table 3 and Pareto analysis of variance (ANOVA). All 3D printing parameters, consisting of nozzle temperature, print speed, infill density and layer height, were set at three different levels, while HNT content comprised six different levels accordingly. The remaining 3D printing parameters were fixed according to Table 4. For simplicity, it was assumed that minimal factorial interaction was taken into account in this design of experiment (DoE) work.

**Figure 3:**
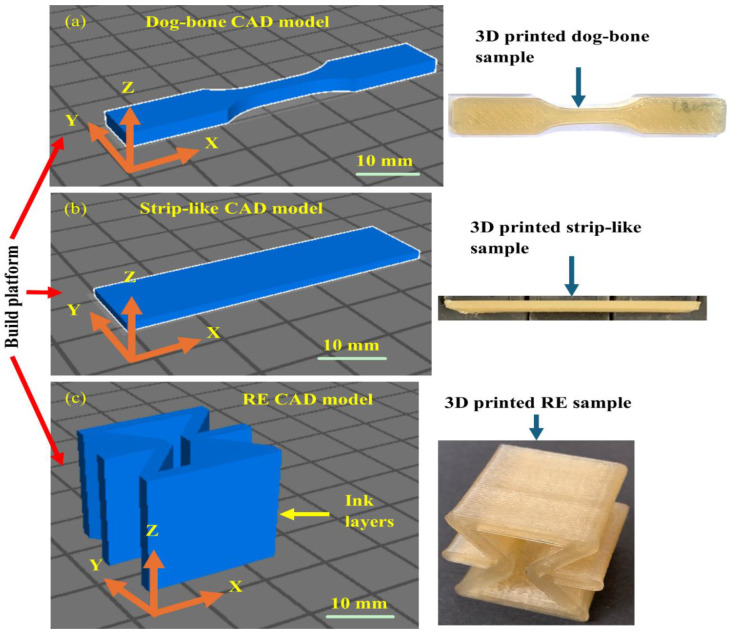
CAD models on 3D printing build platform and final 3D printed (**a**) dog-bone, (**b**) strip-like and (**c**) RE structures.

3D printed PU/HNT nanocomposite samples at HNT contents of 0, 2, 4, 6, 8 and 10 wt% in dog-bone and strip-like forms, based on ASTM D638 (type V) standard [36] and ASTM D790-17 standard [37], as well as RE structures, were then manufactured to determine mechanical and shape memory properties of additively manufactured PU/HNT nanocomposites. PU/HNT nanocomposites at different HNT contents of 0, 2, 4, 6, 8 and 10 wt% are represented by PU, NC-2, NC-4, NC-6, NC-8 and NC-10 respectively. Dog-bone samples, according to ASTM D638 standard (type V), were used in this study since PU MM4520 grade is relatively flexible when compared with polylactic acid (PLA) and acrylonitrile butadiene styrene (ABS). As this study used dog-bone samples based on ASTM D638 standard (type IV), the available extensometer could not measure the elongation at break for such samples in the tensile tests. In addition, the size effect in 3D printing induced by PU is less problematic than PLA. CAD models on the 3D printing build platform, as well as final printed dog-bone, strip-like and RE samples, are depicted in Figure 3a–c. It is worth mentioning that all samples were printed along the *x*-direction for simple analysis.

### 2.3. Tensile Tests

**Figure 4:**
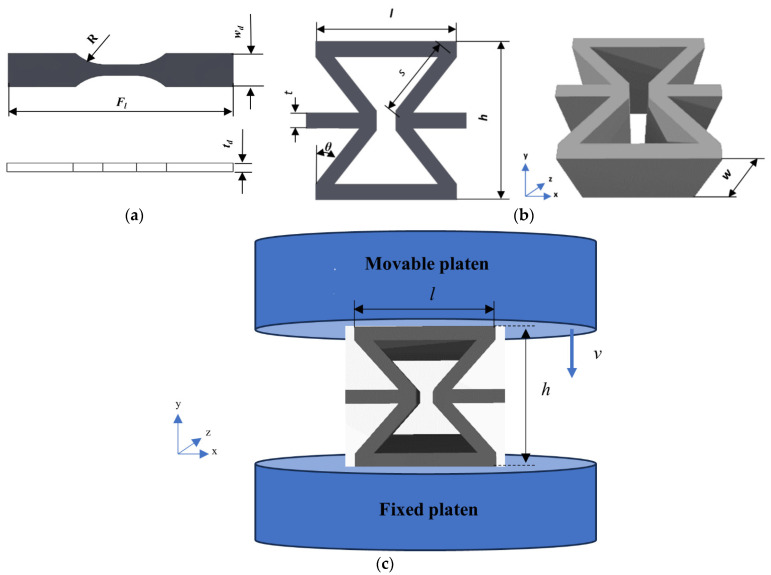
(**a**) A dog-bone structure with reference to ASTM D638 (type V), (**b**) RE structure with its dimensions, (**c**) a schematic diagram of compressive test for RE structure.

A Lloyd universal testing machine EZ50T (Lloyd Instruments Ltd., Bognor Regis, UK), combined with a load cell of 10 kN and a crosshead speed of 5 mm∙min^−1^, was employed to evaluate the impact of HNT content on tensile properties of additively manufactured PU/HNT nanocomposites, consisting of tensile strength at yield, tensile modulus and elongation at break. ASTM D638 (type V) standard was used as a reference to manufacture dog-bone samples, which were then tested at room temperature, as shown in Figure 4a. The radius of curve section (*R*), full length (*F_l_*), thickness (*t_d_*) and width (*w_d_*) of such samples are 12.7, 63.5, 3.18 and 9.53 mm respectively. Five samples were used in tensile tests at each HNT content.

### 2.4. Compressive Tests

The influence of nanofiller content on compressive modulus, compressive strength, Poisson’s ratio (*PR*), as well as specific energy absorption (*SEA*) of PU/HNT nanocomposites was investigated using compressive tests. In relation to this, a RE structure with the length of the inclined strut (*s*) of 12 mm, the length of the vertical strut (*l*) of 20 mm, and the width (*w*) of 20 mm, the height (*h*) of 20 mm, the cell thickness (*t*) of 2 mm and the angle of the inclined strut (*θ*) of 40°, was employed as a typical specimen, as displayed in Figure 4b. The uniaxial quasi-static compressive tests with the in-plane *y* direction were carried out with the same machine and load cell used for the tensile tests at room temperature according to Figure 4c. The sample was set up on the fixed platen of the machine, where three samples were compressed with each HNT content for test reproducibility.

The head displacement rate (*v*) was implemented to a movable top platen, which travelled downwards with a constant speed of 0.5 mm/min based on ASTM C365 standard [38]. Once densification had taken place, the compressive test was terminated. Nominal stress (*σ*) and strain (*ε*) can be defined by applying Equations (1) and (2).

(1) $$\sigma =\frac{F}{l\times w}$$

(2) $$\varepsilon =\frac{\delta }{h}$$

where *l*, *w* and *h* denote the length of the vertical strut, the width and the height of RE structure. On the other hand, *F* and *δ* represent an imposed force, also known as load, as well as a displacement change between the platens of the compressive machine. The data concerning force, instantaneous time and displacement were recorded by a computer affiliated with the machine [39].

The results of the compression test were employed to plot *σ*-*ε* diagrams. The maximum compressive strength of 3D printed nanocomposites was defined by taking into account the greatest stress on compressive testing curves obtained. It is noted that densification strain (*ε_d_*) should be determined before calculating *SEA*. In addition, energy absorption efficiency *η*(*ε*) must be calculated using Equation (3) prior to determining *ε_d_*, as recommended by Dong et al., Shen et al. and Onck et al. [40,41,42].

(3) $$\eta \left(\varepsilon \right)=\frac{\int_{0}^{\varepsilon_{a}}\sigma \left(\varepsilon \right)d\varepsilon }{{\sigma (\varepsilon }_{a})}\;\;\;\;\;\;\;\;\;$$

*ε_a_* is the strain at instantaneous stress (*σ*), while *η* represents the region under the stress-strain curve, which is divided by *σ*. To define *ε_d_*, *η* versus *ε* should be plotted, where *ε_d_* denotes the strain level corresponding to the maximum *η*, which can be written as

(4) $$\frac{d\eta (\varepsilon_{a})}{d\varepsilon }\left|\varepsilon_{a}=\varepsilon_{d}=0\right.$$

SEA is defined as the accumulative region under the stress-strain curve from 0 to *ε_d_*_,_ which is divided by the density (*ρ)* of RE structure according to Equation (5). First of all, the trendline equation was calculated, which was then integrated to determine the accumulative area under the curve using MATLAB R2023a. Ingrole et al. [43] and Bitzer [44] mention that *ρ* could be determined by applying Equation (6) where *t*, *l* and *s* are the cell thickness, the length of vertical strut and the length of inclined strut in RE structures. Additionally, *ρ_s_* signifies the density of bulk material. The densities of bulk material for virgin PU and PU/HNT nanocomposites were determined by employing the density kits with the aid of Equation (7). According to Equation (6), *θ* value for a unit cell of RE structure is negative [43].

(5) $$SEA=\frac{\int_{0}^{\varepsilon_{d}}\sigma \left(\varepsilon \right)d\varepsilon }{\rho }$$

(6) $$\frac{\rho }{\rho_{s}}=\frac{\frac{t}{s}(\frac{l}{s}+2)}{2cos\theta (\frac{l}{s}+sin\theta )}$$

(7) $$\rho =\frac{A}{A-B}\left(\rho_{0}-\rho_{L}\right)+\rho_{L}$$

Poisson’s ratio (PR) can be represented as the negative ratio of the lateral strain to the axial strain. In relation to this, the axial strain was determined according to the displacement of the platen in the *y*-axis. Conversely, a digital Fujifilm X-T3 camera manufactured by Fujifilm Corporation, Japan, was applied to identify RE structure displacement in the *x*-axis so that it may determine the lateral shifting in the *x*-axis using DaVinci Resolve 18 and ImageJ 153 software. Meanwhile, the axial strain (*ε*) and lateral (*ε_L_*) strain were calculated with the aid of Equations (8) and (9) respectively. Here, *h* and ∆*h* represent initial height and height change. Meanwhile, *l* and ∆*l* denote initial length and length change accordingly. PR can be then defined using Equation (10), as recommended by Chow et al. [45].

(8) $$\varepsilon =\frac{\Delta h}{h}$$

(9) $$\varepsilon_{L}=\frac{\Delta l}{l}$$

(10) $$PR=-\frac{\varepsilon_{L}}{\varepsilon }$$

### 2.5. Shape Memory Tests

This study performed two stages of shape memory tests, consisting of preliminary and primary tests. The first stage covered three-point bending tests, along with recovery processes, in order to investigate shape recovery ratio and recovery time for deformed PU/HNT nanocomposite structures. The results of such tests were applied to determine the efficacious temperature for heating sample structures throughout compression tests and recovery processes. Afterwards, primary tests adopted such temperature levels accordingly. Strip-like structures, with the length of 50.8 mm, the width of 12.7 mm and the thickness of 1.5 mm based on ASTM D790-17 standard, were made as three-point bending test samples (see Figure S1a,b in Supporting Information). These tests were not used to determine flexural strength, but instead to prepare the samples in order to study their shape memory behaviour. Regarding this, a UTM-25 universal testing machine integrated with an oven and supplied by IPC Global, Italy, was used to carry out three-point bending tests using a load cell of 25 kN at the crosshead speed of 1 mm/min.

3D printed PU/HNT nanocomposite samples were bent at 50 °C, which was slightly above *T_g_* of PU at 41 °C. Once heated above 50 °C, these samples encountered twisting effect. A digital Digitech QM1601 thermometer, integrated with a K-type thermocouple manufactured by Digitech, China, was applied to determine the sample temperature. A LABEC convection oven, supplied by Laboratory Equipment Pty. Ltd., Australia, was employed to heat nanocomposite samples during the recovery process. In addition, Fujifilm X-T3, a digital camera made by Fujifilm corporation, was implemented to visualise the shape recovery process. Three temperature levels of 60, 70 and 80 °C were selected for a recovery purpose. For each material batch, three samples were investigated to evaluate their shape memory properties for data reproducibility.

According to ASTM D790-17 standard, once the deflection of the midspan (*D*) achieves a determined value with reference to Equation (11), the loading must be terminated. *r*, *L* and *d* represent the strain of 0.05 mm/mm, the support span of 25.4 mm and the depth of beam (or sample thickness) of 1.5 mm. In relation to this, the loading was terminated when *D* was 3.58 mm. Conversely, shape recovery ratio for nanocomposite samples, represented by *R_rs_* and indicated by angle recovery, can be evaluated by using Equation (12). *θ*_0_ and *θ_t_* denote a recovery angle and an angle of programmed bending. The former is assessed when the recovery process is completed, while the latter is evaluated when the loading process is terminated.

(11) $$D=\frac{rL^{2}}{6d}$$

(12) $$R_{r}=\frac{\theta_{0}-\theta_{t}}{\pi -\theta_{t}}\times 100\%$$

RE nanocomposite samples were employed in the primary tests. Concerning this, the dimensions of the samples and the steps of compressive tests to investigate shape memory properties of virgin PU and PU/HNT nanocomposites were combined to those conducted in compression tests using RE structures at room temperature, as shown in Figure 4c, despite using distinct sample temperatures and testing machines. In addition, compression tests were performed at 50 °C using an asphalt mixture performance tester (AMPT) equipped with a load cell of 15 kN. This testing machine possesses a smaller load cell compared to a UTM-25 machine, signifying that AMPT results in more precise data relating to polymers and polymer composites. Additionally, it has a smaller oven, allowing this machine to achieve the desired temperature more rapidly than a UTM-25 machine. Nevertheless, AMPT is not allowed to be employed to perform three-point bending tests owing to environmental chamber size. The tests were terminated when *δ* reached 5 mm. This value was chosen since the onset densification of RE nanocomposite samples took place when δ was achieved at approximately 8 mm.

**Figure 5:**
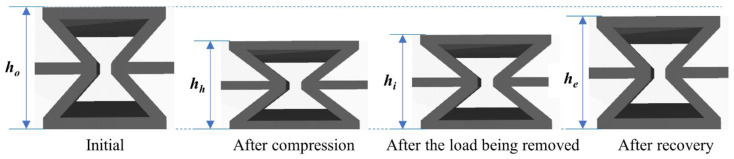
Dimensional changes of RE sample during a shape memory test.

For a recovery purpose, primary characterisation employed a similar oven to the one used in prior tests, and the samples were heated to 80 °C since it resulted in quick recovery due to twisting effect. The heights of RE nanocomposite samples prior to compression, after compression, after the load being eliminated and after recovery are represented by *h_o_*, *h_h_*, *h_i_* and *h_e_*, which are displayed in Figure 5. Conversely, the maximum strain, the amount of deformation after load removal, the amount of reversible strain, the shape fixity ratio and the shape recovery ratio are annotated by *ε_m_*, *ε_u_*, *ε_p_*, *R_f_*, and *R_r_* and calculated using Equations (13)–(17). Three samples were implemented to gain the average data with respect to shape memory properties for test reproducibility. All the required samples for material characterisation for each material composition are listed in Table 5.

(13) $$\varepsilon_{m}=\frac{h_{o}-h_{h}}{h_{o}}$$

(14) $$\varepsilon_{u}=\frac{h_{o}-h_{i}}{h_{o}}$$

(15) $$\varepsilon_{p}=\frac{h_{o}-h_{e}}{h_{o}}$$

(16) $$R_{f}=\frac{\varepsilon_{u}}{\varepsilon_{m}}\times 100\%$$

(17) $$R_{r}=\frac{\varepsilon_{m-}\varepsilon_{p}}{\varepsilon_{m}}\times 100\%$$

It can be seen in Table 5 that this study implemented five replications for the tensile tests and three replications for the other tests. In general, five replications could yield better results. However, even though this study only ran three replications for most mechanical and shape memory tests, it can still represent adequate results, as indicated in forthcoming results of three-point bending tests, compressive tests and shape memory tests with reasonable error bars.

### 2.6. Material Characterisation

Surface microcracks of RE nanocomposite samples were assessed via scanning electron microscopy (SEM) by implementing a Clara field emission scanning electron microscope (FESEM) provided by Tescan GmbH, Germany. For SEM imaging analysis, the voltage and current of the electron beam were set to 5 kV and 43 pA. Afterwards, the samples were placed on a sample holder (diameter: 45 mm) mounted to the electron microscope with double-sided carbon tape. For good conductivity reasons and to avoid any charging effect for better image clarity, this study implemented sputter coating on nanocomposite samples by employing carbon layers (layer thickness: 20 nm).

HNT dispersion in PU/HNT nanocomposites was assessed by an FEI Talos FS200X G2 transmission electron microscope equipped with a field emission gun (TEG). Ultrathin TEM samples with an average thickness of 100 nm were prepared using an ultramicrotome Leica EM UC6 and a glass knife. Afterwards, these samples were put on 300-mesh copper grids for subsequent TEM analysis.

### 2.7. Statistical Analysis

This study implemented Taguchi method with an associated orthogonal array (OA) to create DoE layout and evaluate the influences of factor-level combinations of input factors on output factor, namely tensile strength as the DoE response since it is critical for excellent material performance and the load-bearing capacity of a structure. Therefore, the optimum factor-level combination with respect to maximum tensile strength was chosen as the preferred 3D printing parameter. Meanwhile, ANOVA was used to understand the contribution of each input factor.

Taguchi method mainly relies on the orthogonal matrix of experimental design. The experimental design can be defined as a particular orthogonal array, allowing for the co-occurring impact of several input process parameters to be assessed expeditiously [46]. The objective functions of Taguchi method consist of three criteria, namely, “smaller-the better”, “larger-the better” and “nominal-the better” criteria. The “smaller-the-better” can be chosen to minimise output factors, such as dimensional errors, surface roughness and abrasion loss of 3D printed structures. In contrast, “larger-the-better” and “nominal-the better” criteria can be applied to maximise output factors, namely toughness, hardness and tensile properties of nanocomposite samples. The optimum level for each element is reflected by the level leading to the greatest sum of signal-to-noise (*S*/*N*) ratio within experimental design. In this study, *S*/*N* ratio was used to determine the response sensitivity in a controlled manner in relation to uncontrolled external noise factor. Equation (18) was employed to determine *S*/*N* ratios for the “larger-the-better” criterion, as suggested by Ross [47].

(18) $$S/N_{i}=-10{Log}_{10}\frac{1}{n_{i}}\left(\sum_{u=1}^{n_{i}}\frac{1}{y_{u}^{2}}\right)$$

where *i*, *u*, *n_i_* and *y_u_* denote the number of experiments, the trial number, the number of trials for *i*th experiment and the data observed as the output response. A greater *S*/*N* ratio results in a better result since it yields better quality with minimum variance. Consequently, the greatest sum of *S*/*N* ratio is key to reaching the optimum factor-level combination.

The contribution of each 3D printing input parameter on observed output responses (i.e., the tensile properties of the samples) was assessed based on ANOVA [48]. This method does not require an ANOVA table and *F*-tests to analyse final results for parametric design [49] Consequently, it is less time-consuming for data analysis. The sum of squares (*SSs*) and the contribution of each factor (*P*) can be calculated using Equations (19) and (20) [47].

(19) $$SS_{A}=\left[\sum_{i=1}^{k_{A}}\left(\frac{A_{i}^{2}}{n_{A_{i}}}\right)\right]-\frac{T^{2}}{N}$$

(20) $$P_{A}=\frac{SS_{A}-(\nu_{A}\times V_{e})}{SS_{T}}\times 100\%$$

where *SS_A_*, *A* and *n_A_* represent the sum of squares, the value and number of observations of factor A. *T* and *N* are the sums of all observations and the total number of observations respectively. Meanwhile, the contribution percentage, the degree of freedom, the variance due to error and the total sum of the squares of factor A (nozzle temperature) are indicated by *P_A_*, *v_A_*, *V_e_* and *SS_T_*_._ On the other hand, *SS* and *P* values for other factors, including factors B (infill density), C (print speed) and D (layer height), can be calculated using Equations (19) and (20). The degree of freedom (dof) of each factor *ν_i_* can be defined as a total of the levels subtracted by 1.

## 3. Result and Discussion

### 3.1. Tensile Properties

**Figure 6:**
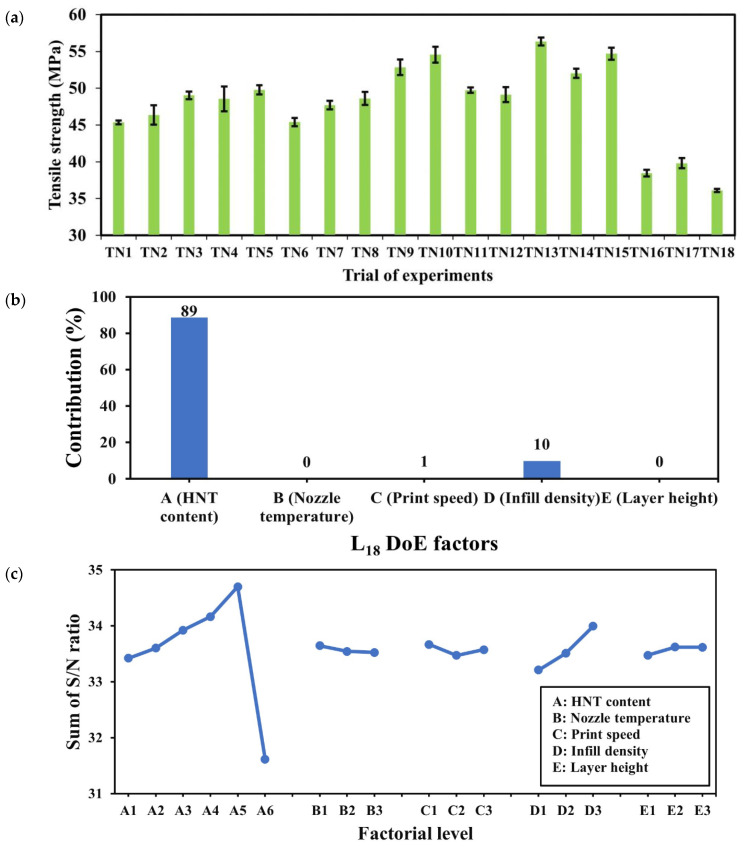
(**a**) Tensile strength, (**b**) Pareto ANOVA and (**c**) sum of *S*/*N* ratios for 3D printed PU/HNT nanocomposite dog-bone samples based on *L*_18_ layout in DoE work [35].

As seen in Figure 6a, the tensile strengths of PU/HNT nanocomposite samples appeared to increase when HNT content increased up to 8 wt%, where the ultimate tensile strength reached approximately 56.34 ± 0.556 MPa for TN13, which was 24.2% greater than that of TN1 (i.e., 0 wt% HNTs) at 45.35 ± 247 MPa. This phenomenon signifies a good interaction between PU molecular chains and HNTs that potentially increased hydrogen bonding due to the presence of carboxyl groups on HNT surfaces, which is in good accordance with Mahunaki et al. [27]. However, the tensile strength suddenly dropped when HNT content continued to increase up to 10 wt% because of typical nanofiller agglomeration. Consequently, the lowest tensile strength of 36.11 ± 0.223 MPa was gained for TN18 owing to weak filler–matrix interaction.

Factorial contribution with respect to tensile properties of dog-bone samples made from PU/HNT nanocomposites was calculated using Pareto ANOVA. As mentioned earlier in Section 2.7, significant factors can be determined when their cumulative contribution exceeds 90% [49]. It is clearly indicated in Figure 6b that HNT content (factor A), and infill density (factor D) significantly affected tensile strength with a cumulative contribution of 99%. The incorporation of HNTs and the presence of air gaps between their 3D printed layers tend to reduce the tensile strength of nanocomposite samples according to Chie et al. [50]. In contrast, the effect of nozzle temperature (factor B), print speed (factor C) and layer height (factor E) was quite negligible as non-significant factors in this study notwithstanding that Vidakis et al. [51] mentioned that increasing printing temperature and layer height might generate an adverse impact on tensile strength.

The “larger-the-better” criterion was used in Taguchi DoE analysis to assess the optimum factor-level combination so that it could maximise mechanical properties. A higher sum of *S*/*N* ratios typically results in a better response for the factorial effect. Figure 6c shows that increasing HNT content up to 8 wt% increased the sum of *S*/*N* ratios. Nevertheless, it dramatically dropped when HNT content reached 10 wt% due to HNT agglomeration. Infill density was considered the second significant factor with respect to the maximisation of tensile strength. This study estimated that maximum tensile strength might be obtained at the infill density of 100% for solid material samples, which is in good agreement with Wang et al. [52]. In addition, it was found that negligible influence was imposed by other input factors, such as nozzle temperature, layer height and print speed. Overall, the optimum factor-level combination with respect to maximum tensile strength was achieved at HNT content of 8 wt%, nozzle temperature of 210 °C, print speed of 10 mm/s, infill density of 100% and layer height of 0.4 mm, which can be referred to as A_5_B_1_C_1_D_3_E_3_ according to Table 6. Meanwhile, other parameters, such as quality, fill, temperature, travel speed, filament flow and retraction speed, should be fixed, as shown in Table 4.

**Figure 7:**
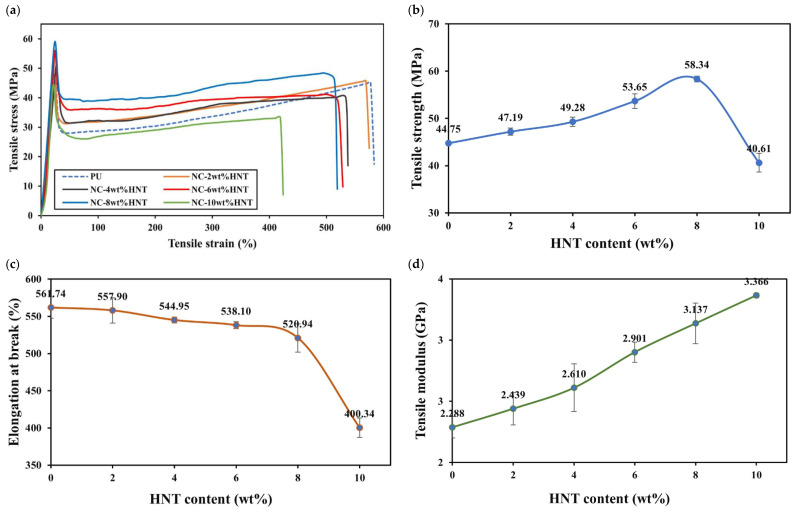
(**a**) Stress–strain curve and influence of HNT content on (**b**) tensile strength, (**c**) elongation at break and (**d**) tensile modulus of PU/HNT nanocomposites.

**Figure 8:**
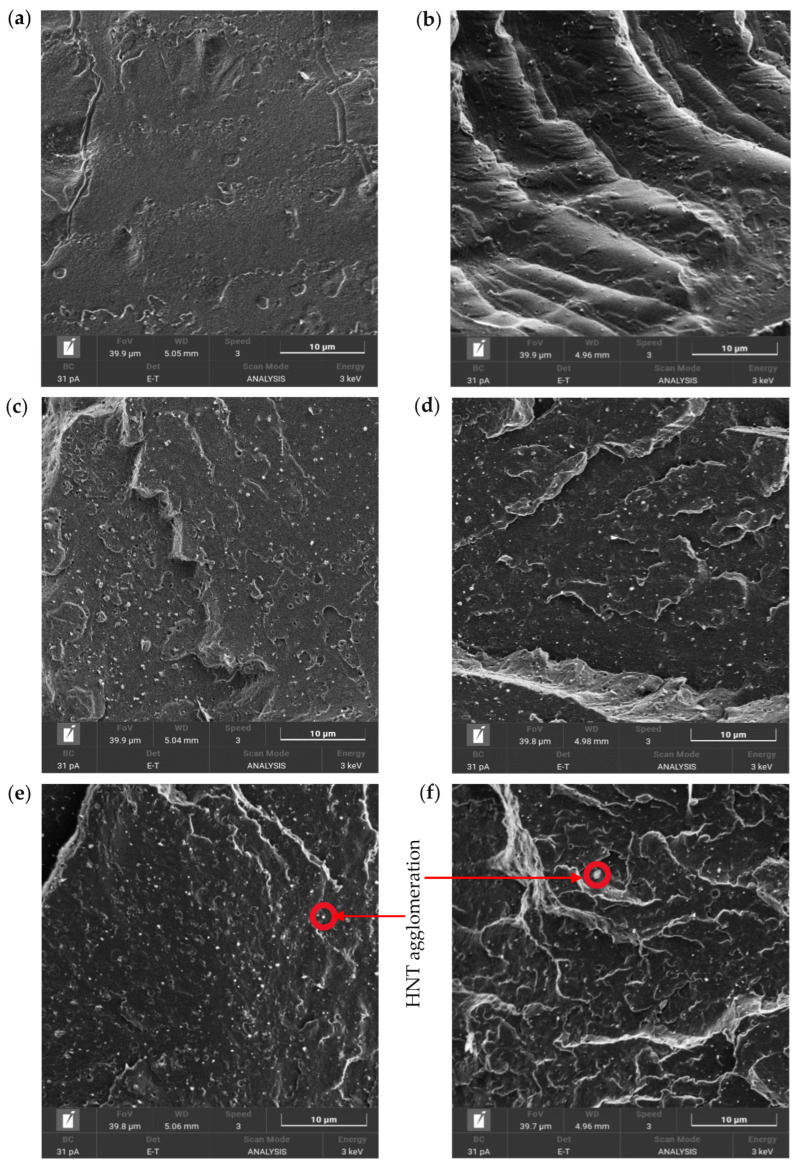
SEM micrographs of (**a**) PU and PU/HNT nanocomposites at different HNT contents: (**b**) 2 wt%, (**c**) 4 wt%, (**d**) 6 wt%, (**e**) 8 wt% and (**f**) 10 wt%.

**Figure 9:**
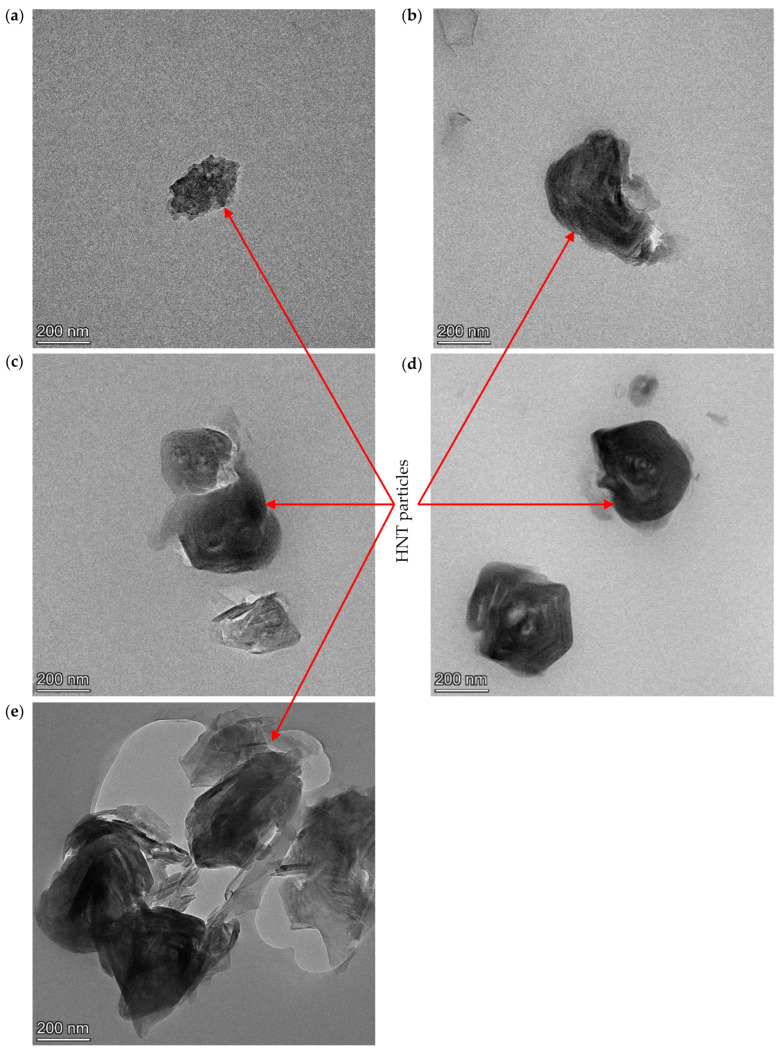
TEM micrographs showing HNT dispersion in PU/HNT nanocomposites at different HNT contents: (**a**) 2, (**b**) 4, (**c**) 6, (**d**) 8 and (**e**) 10 wt%.

Typical stress–strain curves and corresponding mechanical properties, such as tensile modulus, tensile strength and elongation at break, for PU/HNT nanocomposites manufactured according to the parametric settings in Table 6 at different HNT contents, are depicted in Figure 7a–d. In general, the inclusion of HNTs improved the tensile strength of 3D printed PU/HNT nanocomposites (Figure 7a). The addition of 8 wt% HNTs could increase the tensile strength of nanocomposites by 30.4% relative to that of virgin PU at 44.75 ± 0.431 MPa, as shown in Figure 8b. This might be associated with better filler–matrix interaction due to the presence of surface functional groups, as reported earlier by Mahunaki et al. [27]. As such, higher energy was demanded in order to stretch PU/HNT nanocomposites when compared with virgin PU. However, when HNT content reached 10 wt%, it reduced the tensile strength of nanocomposite samples to 40.61 ± 1.990 MPa because of typical HNT agglomeration. This finding could arise from poor filler–matrix interaction, as indicated through SEM observation shown in Figure 8. Figure 8a presents the microstructure on neat PU surface. HNTs were clearly well dispersed within PU matrices, which was particularly the case for low HNT contents of 2, 4 and 6 wt% according to Figure 8b–d. However, some localised HNT agglomeration occurred at higher HNT contents of 8 and 10 wt%, as illustrated in Figure 8e,f. These results are well supported by corresponding TEM micrographs presented in Figure 9a–e.

In contrast, increasing HNT content tended to reduce the flexibility of PU/HNT nanocomposites, leading to their reduction in elongation at break. Figure 7c shows an abrupt drop occurred at the HNT content above 2 wt%. Generally speaking, the inclusion of rigid nanofillers typically promotes the brittle nature of polymer composites. The minimum elongation at break took place with the inclusion of 10 wt% HNTs, which is equal to a 28.7% drop relative to that of virgin PU.

As for tensile strength, the incorporation of HNTs within PU matrices also increased tensile moduli of PU/HNT nanocomposites, as illustrated in Figure 7d. The largest tensile modulus of 3.37 ± 0.018 GPa was reached with the inclusion of 10 wt% HNTs. At this HNT content, the tensile modulus was increased by 47.2% when compared with that of neat PU at 2.29 ± 0.088 GPa. This might be correlated with the stiffening effect of rigid nanofillers, which could further limit the chain movement of PU molecules. Consequently, the flexibility and extensibility of virgin PU declined when enhancing nanocomposite stiffness, as mentioned by Sulong et al. [29]. Generally speaking, tensile properties of nanocomposite samples achieved in this study are in good agreement with the results reported by Namathoti et al. [53].

### 3.2. Compressive Properties

**Figure 10:**
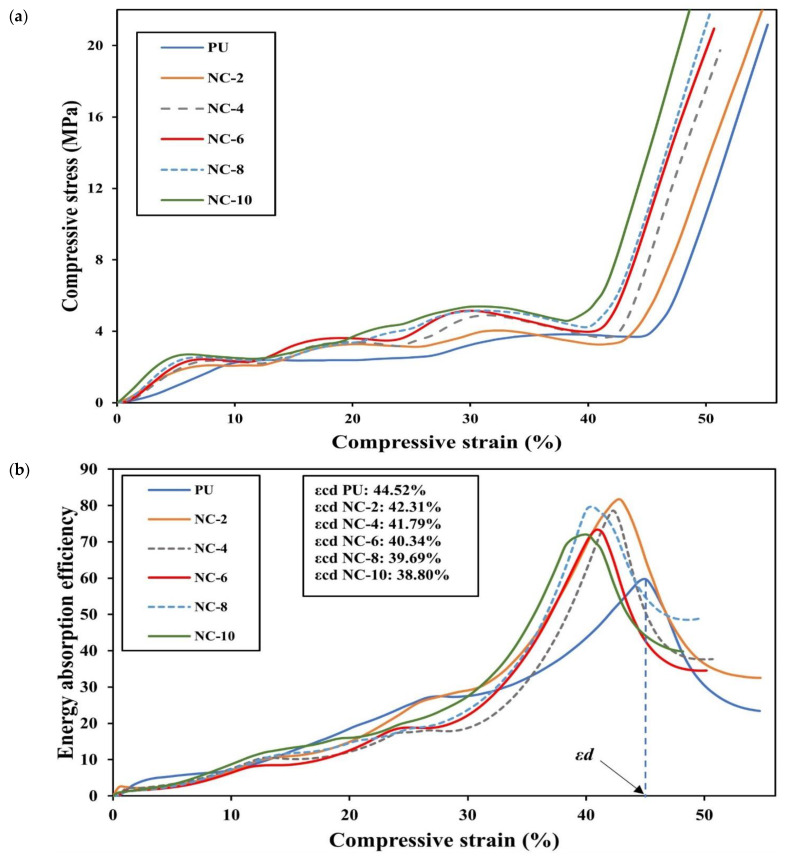
(**a**) compressive stress-strain curves and (**b**) energy absorption efficiency curves of RE structures.

The incorporation of HNTs within PU matrices enhanced the compressive strength of RE structures, as depicted in Figure 10a. The resulting curves possessed three regions comprising elastic, plastic and densification zones. It is clearly seen that a greater HNT content shifted up the curve, indicating a good impact of the reinforcement on improving the compressive strength of 3D printed PU/HNT nanocomposites. *ε_d_* values, indicating onset strain densification of PU/HNT nanocomposites, were lower than that of virgin PU. Nanocomposite samples with an HNT content of 10 wt% possessed *ε_d_* of approximately 38.80% when compared with that of virgin PU at 44.52% according to Figure 10b. This result suggests that the presence of HNTs increased the stiffness of PU/HNT nanocomposite samples, thus accelerating material densification.

Trendline functions in relation to compressive stress-strain curves of PU/HNT nanocomposites at all HNT contents and the associated integration results for those trendlines are presented in Table 7 and Table 8. The *R^2^* values for determined trendline functions exceeded 0.95. This implies that the variation in the dependent variable (i.e., compressive stress), as indicated by an independent variable (i.e., compressive strain), is classified as a good fit (Table 7). Subsequently, such trendline functions were integrated with the aid of MATLAB R2023a, and the integration results are presented in Table 8. These integration results were used to determine energy absorption efficiency according to Equation (3). Subsequently, they were applied to calculate SEA based on Equation (5), along with the requirement of density values for PU/HNT nanocomposites. These densities were obtained using density kits based on Equations (6) and (7), as listed in Table 9. The average density of virgin PU was determined to be 1200 kg/m^3^, which was slightly lower than that mentioned by the manufacturer (i.e., 1250 kg/m^3^) [32]. It can be clearly seen from Table 9 that an increase in HNT content up to 10 wt% resulted in a minor impact on the density increase of RE structures by approximately 1.83%. Meantime, the influences of HNT content on compressive properties and SEA of PU/HNT nanocomposites are displayed in Figure 10a,b.

In general, the inclusion of HNTs into PU matrices increased the compressive strength of PU/HNT nanocomposites, mainly with the addition of 4 wt% HNTs, implying a positive reinforcing impact of these nanofillers (see Figure S2a in Supporting Information). Further increasing the nanofiller content only slightly enhanced the compressive strength. The highest compressive strength of PU/HNT nanocomposites (i.e., 5.20 MPa) was obtained at the highest nanofiller content (i.e., 10 wt% HNTs), as opposed to that of virgin PU (i.e., 3.88 MPa). It was mentioned by Nie et al. [54] that a PU sponge that was coated with polydopamine possessed a compressive strength of approximately 1.26 kPa at 50% strain, which was 400% greater than an untreated PU sponge. This might be associated with the effect of covalent linking that occurred between these composites and γ-aminopropyltriethoxysilane that grafted to HNTs, leading to an increase in the rigidity of sponge skeleton. Another study by Lorusso et al. [55] revealed that HNT inclusion up to 10 wt% within PU matrices improved the compressive strength from 96.84 to 106.56 MPa for foam structures made of PU/HNT nanocomposites.

Similar to compressive strength, the increase in nanofiller content monotonically improved the compressive modulus of nanocomposite samples, as indicated in Figure S2a in Supporting Information. The incorporation of HNTs within polymeric matrices, such as PU, can elevate the higher level of phase separation in hard segments of PU through the hydrogen bonding existing in urethane groups and the contact of hydroxyl groups to HNT surfaces, according to Mahunaki et al. [27]. This phenomenon resulted in a greater hydrogen bonding density in hard segments in order to hinder the movement of polymeric chains, leading to higher stiffness [31]. It can be induced by the presence of aluminol and siloxane groups on HNT surfaces, thus facilitating the formation of hydrogen bonding with PU molecular chains onto their surfaces. Accordingly, the compressive modulus of 3D printed nanocomposites with the addition of 10 wt% HNTs was improved up to 1.26 folds when compared with that of virgin PU, according to Figure S2a in Supporting Information. It has been found by Salaman et al. [56] that HNT addition up to 3 wt% within PU matrices improved the compression modulus of nanocomposites by 1.67 GPa, as opposed to that of virgin PU at 1.29 GPa.

Similar to compressive strength and compressive modulus, the presence of HNT particles within PU matrices increased the density of PU/HNT nanocomposites with their capacity to bear a greater load than virgin PU. Accordingly, an increase in HNT content increased SEA of nanocomposites, as depicted in Figure S2b in Supporting Information. The greatest SEA of 0.76 kJ/kg was obtained at HNT content of 10 wt% when compared with that of virgin PU (i.e., 0.44 kJ/kg). The resulting data are supported by El-baky et al. [57], which used HNTs as the additives to enhance the performance of crashworthiness for tubular structures based on epoxy/glass composites. It was reported that the tubes, which were unfilled, obtained SEA about 11.50 J/g. Meanwhile, the presence of HNTs at different contents of 1, 2, 3 and 4 wt% induced notable improvements for SEA of nanocomposite tubes up to 160.61%, 215.30%, 190.96% and 233.91%. This improvement might be correlated with toughening effect of HNTs in relation to front pinning, along with deflection and the bridging of resulting cracks [57]. Once microcracks subjected to the axial load in quasi-static conditions met rigid nanofillers like HNTs, these cracks were diminished by the crack-bridging phenomenon of these nanofillers, as mentioned earlier by Ye et al. [58] when further reinforcing epoxy/carbon fibre composites. A similar phenomenon was also identified by Silva et al. [59] for other embedded additives, like nano-silica and MMT, as well as glass-sphere particles used as the secondary fillers for polyamide 6/glass fibre composites. A summary of compressive properties for 3D printed nanocomposites, comprising compressive strength, compressive modulus and SEA, is provided in Table 10.

**Figure 11:**
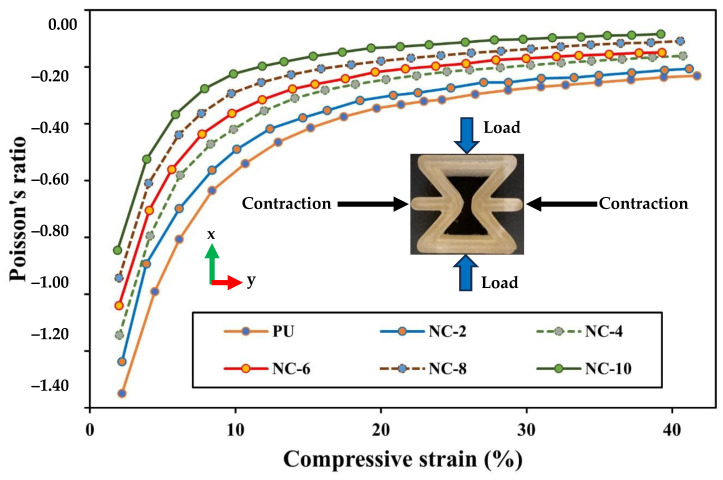
Effect of HNT addition on PRs for RE structures.

The inclusion of HNTs within PU matrices hindered polymeric chain movement, resulting in much higher stiffness. The presence of aluminol and siloxane groups on HNT surfaces generates hydrogen bonding with PU molecular chains, inducing their movement restriction. Furthermore, the increase in HNT content tended to lower engineering strain mainly in the lateral direction, which led to a reduction in NPRs according to Figure 11. PU/HNT nanocomposite RE structures possessed an auxetic characteristic owing to their particular structures, allowing them to encounter transverse contraction when subjected to uniaxial compression [16]. At 10% compression strain, the curve parts of the strut in the middle area of RE structure touched each other, followed by less lateral strain. It was clearly indicated that the trend of all curves was consistent from initial sharp increases to near plateaus, in good accordance with Park et al. [60], which evaluated the efficacy of chondrocyte proliferation for PU auxetic scaffolds under compressive tests, particularly applicable to tissue engineering of articular cartilage. In general, PR versus compressive strain curves for PU/HNT nanocomposites are in good agreement with Alomarah et al. [39], where PR values were determined according to engineering lateral strain and axial strain for the samples under compression in the *x*-direction.

### 3.3. Shape Memory Properties

Shape memory properties in certain polymers are influenced by microphase separation of hard and soft segments, according to Abdullah et al. [61]. During a programming step, soft segment phases fixed deformed hard segment phases after nanocomposite strip-like samples were bent at the elevated temperature of 50 °C (i.e., above their *T_g_*). Afterwards, they were cooled to room temperature (i.e., below their *T_g_*). The induced stress throughout this step was kept in the cross-linked hard segments. It was further set free after being heated during the recovery step.

Generally speaking, HNTs usually absorb hard segments in the soft domain, and thereby only soft segments have a significant influence on shape recovery and original form features [62]. Increasing HNT content tends to cause HNT agglomeration, which makes the movement of hard segments highly limited, leading to a decrease in material flexibility [29]. As such, it unavoidably obstructs the recovery process of deformed PU/HNT nanocomposites.

**Figure 12:**
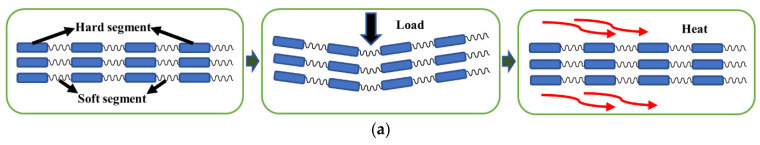

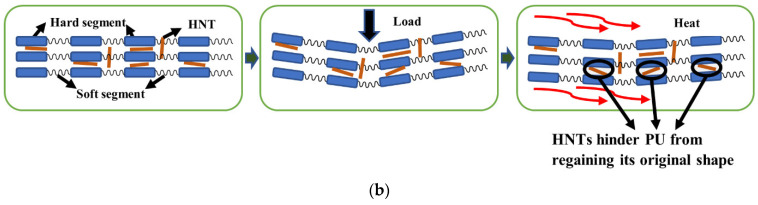
Deformation mechanisms for (**a**) virgin PU and (**b**) PU/HNT nanocomposite samples.

Figure 12 illustrates the processes of applying load and heat as particular stimuli to obtain shape recovery for strip-like nanocomposites. These nanocomposites have molecular structures, consisting of hard and soft segments, as shown in Figure 12a. Subsequently, this structure was deformed via three-point bending tests, in which a load was imposed in the perpendicular direction to the structures. Such deformed structures were heated in the oven, and heat transfer then occurred, driving the movement of hard segments for performing the recovery process. The addition of HNTs within PU matrices prevented the movement of hard segments for recovery according to Figure 12b.

**Figure 13:**
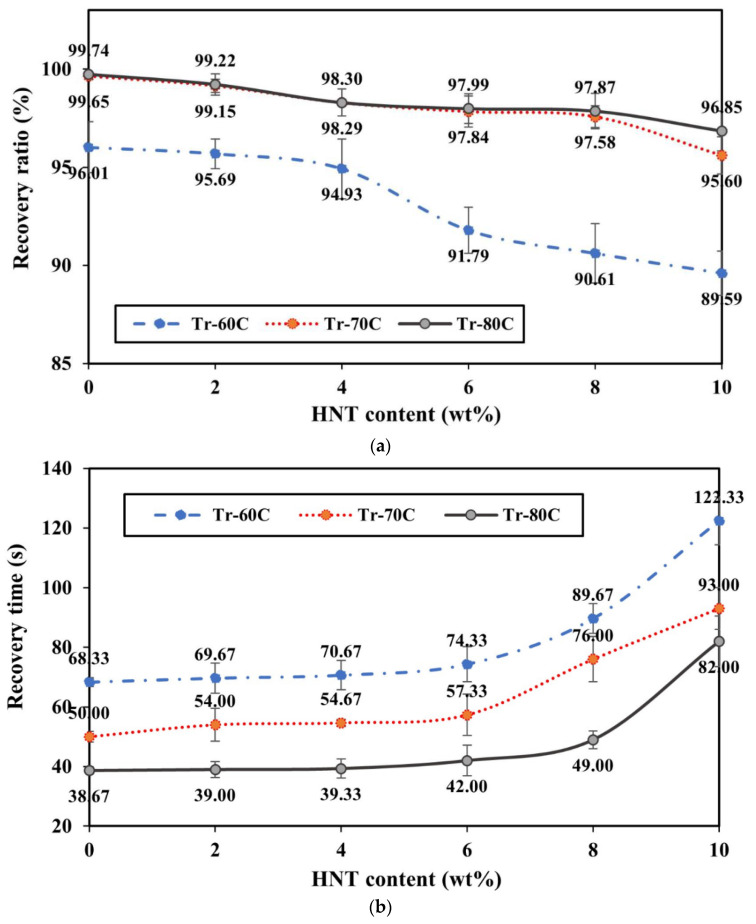
(**a**) Recovery ratio and (**b**) recovery time of strip-like nanocomposites with respect to optimum factor-level combination and ultimate tensile strength.

Figure 13 shows the shape memory properties of strip-like nanocomposite samples at HNT contents of 0–10 wt%. Three typical recovery temperatures (*T_r_*), namely 60, 70 and 80 °C, are represented by Tr-60C, Tr-70C and Tr-80C respectively. It was mentioned earlier that the inclusion of HNTs hindered deformed PU/HNT nanocomposites from regaining their initial shape. As a consequence, the increase in HNT content decreased the recovery ratio, as shown in Figure 13a, in good accordance with Mohammadzadeh et al. [62]. However, dissimilar outcomes were mentioned by Namathoti et al. [53] with the inclusion of 1 wt% HNTs. This study reveals that the increase in HNT content enhanced shape recovery ratio and shortened recovery time. This could be related to disparate fabricating processes (i.e., 3D printing vs. extrusion and injection moulding) diverse processing conditions, as well as nanoparticle content.

Figure 13a shows that recovery temperature significantly influenced shape recovery ratio. In general, higher *T_r_* yields a higher recovery ratio, which might be related to heat transfer from the oven to PU/HNT nanocomposites. The heat allowed the movement of hard segments to recover the initial form of nanocomposites after they were deformed. Once the temperature level of deformed samples was increased up to 60 °C (i.e., Tr-60C), the heat energy provided in this process was not adequate to perform complete shape recovery. The greatest *R_r_* value for virgin PU was only achieved up to 96.01%, along with a slowly declining trend for PU/HNT nanocomposite samples when HNT content was increased. A significant drop in recovery ratio occurred when HNT content exceeded 4 wt%. This could be caused by HNT agglomeration to restrain hard segments of the matrices from regaining their initial form. The minimum recovery ratio of 89.59% was obtained for PU/HNT nanocomposites with the inclusion of 10 wt% HNTs.

Increasing the recovery temperature up to 70 °C, as reflected by the curve of Tr-70C, raised the recovery ratio by 99.65% and 95.60% for virgin PU and PU/10 wt% HNT nanocomposites respectively according to Figure 13a. Heat energy in this heating process enabled the movement of PU molecular chains in nanocomposites, leading to greater recovery when compared with Tr-60C. Further elevating the recovery temperature up to 80 °C (i.e., Tr-80C) slightly enhanced the recovery ratio, especially for nanocomposites with the inclusion of 10 wt% HNTs. Nonetheless, Tr-70C was not able to supply sufficient thermal energy to deal with HNT agglomeration issue that precluded PU from regaining its initial form.

Figure 13b displays the effects of HNT content and recovery temperature on recovery time for nanocomposite strip-like samples. It can be seen that higher HNT contents extended recovery time because of the movement limitation of hard segments within PU matrices when combined with rigid nanofillers. Consequently, a longer recovery time was demanded by nanocomposite samples with higher HNT contents. The longest recovery time of approximately 122 s was achieved at the highest HNT content of 10 wt%. Meanwhile, the shortest recovery time of 68 s was detected for virgin PU at Tr-60C, as shown in Figure 13b. Once *T_r_* of nanocomposites was elevated to 70 °C, recovery time for PU/10 wt% HNT nanocomposites and virgin PU was reduced to 93 and 50 s respectively. The increase in *T_r_* up to 80 °C shortened the recovery time for PU/10 wt% HNT nanocomposites and PU to 82 and 37 s. This could be related to the quantity of heat transfer required by PU to perform shape recovery. A higher *T_r_* supplied more energy to speed up shape recovery of nanocomposite samples, which conversely reduced the recovery time. It could be concluded that 80 °C was an effective heating temperature, which was applied for three-point bending tests and recovery processes in order to characterise shape memory properties of nanocomposite RE structures.

Similar to strip-like nanocomposite samples, HNT content for nanocomposite RE structures was also changed from 0 to 10 wt%. Nevertheless, RE nanocomposite samples were tested using compressive tests instead of three-point bending tests. A temperature level of 80 °C was applied for compression and recovery processes. Furthermore, shape fixity ratio and shape recovery ratio, represented by *R_f_* and *R_r_*, along with recovery time of RE structures, were also discussed in this study. It was aforementioned that the inclusion of HNTs restricted the movement of hard segments and hindered deformed samples from regaining their initial form. It can be said that *R_f_* reflected the capability to change the segments in order to fix compressed height *h_h_* temporarily upon load elimination. Accordingly, nearly all materials possessing shape memory properties can be instantly changed from *h_h_* to *h_i_*. Based on Equation (13), *R_f_* approaches 100% when *h_i_* is close to *h_h_*, as shown in Figure 5.

The temporary or transient shape of nanocomposite RE structures was fixed through crystallisation induced by the strain of soft segments to minimise the temperature of switching transition according to Abdullah et al. [61] When the temperature of PU/HNT nanocomposites is elevated above *T_g_*, compressed and cooled below *T_g_*, soft-segment phase freezes polymeric chains of nanocomposites in their deformed position. Aside from hard segments of crystallites, soft-segment crystallisation is induced by the deformation within the rubbery state throughout the cooling process. It prevents the movement of polymeric chains and restricts strain recovery upon the elimination of applied stress. Therefore, the temporary shape of nanocomposites is maintained when the applied load is eliminated. Nevertheless, Figure S3a in Supporting Information indicates that *R_f_* value of RE nanocomposites without HNTs reached 80.21%, which meant that these nanocomposites were unable to keep their deformed dimension in the vertical orientation (i.e., height) after the load was eliminated. Once the applied load was released, they were instantly changed from *h_h_* to *h_i_*, which demonstrates that soft segments were unable to provide a sufficient barrier to deformed segments for relaxation owing to their partial crystallisation.

The incorporation of HNTs within PU matrices typically elevated *R_f_* value of nanocomposite RE structures because of strain-induced crystallisation effect [60]. The addition of rigid additives like HNTs enhanced soft-segment crystallinity. Moreover, the distribution of strain in PU matrices adjacent to HNTs became larger throughout the deformation process. This might be generated by considerable modulus distinction between the matrices and the reinforcements [63]. Strain localisation could increase the strain-induced crystallisation generated by higher crystallisation that restricted the relaxation process of deformed polymeric chains. Increasing HNT content improved the strain-induced crystallisation, as evidenced by shorter *h_i_*, thus leading to greater *R_f_*.

The potential ability of materials with shape memory properties for recovery is reflected by *R_r_*. RE nanocomposite temperature was enhanced to recover original length *h_0_* from temporary length *h_i_*. In general, shape memory materials enable to regain their height until reaching *h_e_*. During the recovery period, internal stresses generated between hard-segment crystallites that were physically cross-linked and soft-segment crystallites that were stored during the deformation may relax when RE nanocomposites were reheated to exceed their *T_g_*. This process reduced the rigidity of soft segments and enhanced micro Brownian movement [63]. Meanwhile, frozen stress was then active over the shape recovery period. Overall, shape recoverability relies mainly on the capability of glassy hard segments to maintain their original form through either intra- or inter-polymeric chain interactions (i.e., the interactions of dipole–dipole or hydrogen bonding). HNTs, as the additives, can induce the moveability decrease of polymeric chains. Accordingly, the increase in HNT content unavoidably reduced *R_r_*.

As for strip-like nanocomposite structures, the inclusion of HNTs within nanocomposite RE structures restricted the movement of polymeric chains located in hard segments of PU molecules reinforced with HNTs. As a result, a longer recovery time was typically required by RE nanocomposites with higher HNT contents for shape recovery, as presented in Figure S3b in Supporting Information. In addition, nanocomposite RE structures have more intricate geometries, especially in the curved parts, when compared with strip-like nanocomposite samples, leading to a lower shape recovery ratio and a prolonged recovery time. Recovery time appeared to be in a linearly increasing manner from 3.45 to 5.02 min with increasing HNT contents from 0 to 10 wt% since a higher HNT content reduced recovery ability of nanocomposites so that PU/HNT nanocomposites required longer recovery time. A summary of specific shape memory properties for nanocomposite RE structures at different HNT contents is listed in Table 11.

## 4. Conclusions

The effect of HNT addition on mechanical and shape memory properties of additively manufactured PU/HNT nanocomposites, was holistically analysed in this study. The incorporation of HNTs up to 8 wt% within PU matrices greatly increased the tensile strength of nanocomposite samples by 30% when compared with that of virgin PU. A further increase in HNT content up to 10 wt% can improve tensile modulus, compressive strength, compressive modulus and SEA of nanocomposite samples by 47.2%, 34.0%, 125% and 72.7% relative to those of virgin PU. This was induced by the improvement of filler-matrix interaction due to surface functional groups of HNTs and hard segments of PU matrices. When HNT content was increased, the engineering strain of nanocomposite samples, especially the lateral strain, declined because of the impact to limit the movement of polymeric chains, thus decreasing NPR of RE structures. It has been mentioned earlier that PU/HNT nanocomposite RE structures exhibit an auxetic characteristic because of their specific construction, allowing them to undertake transverse contraction when subjected to uniaxial compression. Accordingly, when the HNT content was increased, the lateral strain was decreased, leading to a reduction in NPR of PU/HNT nanocomposites.

The existence of HNTs restricted the movement of polymeric chains, causing various influences on shape memory properties of PU/HNT nanocomposites. First of all, it was related to the improved ability of corresponding nanocomposite samples to be programmed, which enhanced their shape fixity ratio. The addition of HNTs s up to 10 wt% improved the shape fixity ratio of such nanocomposites by 12% relative to virgin PU. The second influence could be associated with the nature of HNTs as rigid nanofillers that obstructed PU matrices from recovering their original form, leading to the reduction of shape recovery ratio. When PU matrices were combined with a high HNT content of 10 wt%, the shape recovery ratio of nanocomposite samples slightly declined by approximately 9%. The last influence is related to the contribution of HNTs as nanofillers to obstruct PU from regaining its initial shape, and further prolonging the recovery time by 45% when using PU/ 10 wt% HNT nanocomposites.

## Figures and Tables

**Table 1:** Physical and mechanical properties of PU MM-4520 grade [32].

Physical Properties		Mechanical Properties		Thermal Properties	
Melt viscosity (Pa·s at 215 °C)	3310	Young’s modulus (MPa)	729	Glass transition temperature (°C)	45
		Tensile strength at break (MPa)	41.4		
		Ultimate elongation (%)	600		
		Shore D hardness	72		

**Table 2:** Properties of HNT particles [33].

Property	Value
Moisture content (%)	3.0
Specific gravity	2.55
pH (aqueous slurry at 20% solids)	3.5–4.5
Surface area [Brunauer–Emmett–Teller (BET)] (m^2^/g)	25
Linear shrinkage (dried at 110 °C) (%)	3.8
Modulus of rupture (dried at 110 °C) (MPa)	2.9

**Table 3:** *L*_18_ OA for PU/HNT nanocomposite samples in DoE study [35].

Exp.		Factor				
	Symbol	A HNT Content wt%	B Nozzle Temperature °C	C Print Speed mm/s	D Infill Density %	E Layer Height mm
1	TN1	0	210	10	40	0.2
2	TN2	0	220	20	70	0.3
3	TN3	0	230	30	100	0.4
4	TN4	2	210	10	70	0.3
5	TN5	2	220	20	100	0.4
6	TN6	2	230	30	40	0.2
7	TN7	4	210	20	40	0.4
8	TN8	4	220	30	70	0.2
9	TN9	4	230	10	100	0.3
10	TN10	6	210	30	100	0.3
11	TN11	6	220	10	40	0.4
12	TN12	6	230	20	70	0.2
13	TN13	8	210	20	100	0.2
14	TN14	8	220	30	40	0.3
15	TN15	8	230	10	70	0.4
16	TN16	10	210	30	70	0.4
17	TN17	10	220	10	100	0.2
18	TN18	10	230	20	40	0.3

**Table 4:** Fixed 3D printing parameters for PU/HNT nanocomposite samples.

Parameter	Specific Parameter	Setting
Quality	Shell thickness (mm)	1.6
	Initial layer thickness (mm)	0.5
	Initial layer line width (%)	120
	Top surface quality	precise
Fill	Bottom/top thickness (mm)	1.2
	Infill interface density	dense
	Infill type	triangle
	Infill overlap (%)	15
Temperature	Bed temperature (°C)	55
Speed	Travel speed (mm/s)	150
	Bottom layer speed (mm/s)	15
	Infill speed (mm/s)	30
Filament	Flow (%)	115
Retraction	Speed (mm/s)	30
	Distance (mm)	5
	Minimum travel (mm)	1.5
	Minimal extrusion before retracting (mm)	0.005

**Table 5:** Tested samples.

Required Tests	Sample Type	Number of Tested Samples
Tensile tests	Dog-bone (ASTM D638 type V)	5
Three-point bending tests	Strip-like (ASTM D790-17)	3
Compressive tests	Re-entrant	3
Shape memory tests	Strip-like (ASTM D790-17)	3
	Re-entrant	3

**Table 6:** The optimum factor-level combination in relation to the maximum tensile strength of 3D printed PU/HNT nanocomposites.

Parameter	Setting
Nozzle temperature (°C)	210
Print speed (mm/s)	10
Infill density (%)	100
Layer height (mm)	0.4

**Table 7:** Best-fit trendline functions in relation to compressive stress–strain curves for PU/HNT nanocomposite samples.

Sample	Trendline Function	R^2^
PU	σ(ε) = 8 × 10^−7^ ε^5^ − 9 × 10^−5^ ε^4^ + 3.9 × 10^−3^ ε^3^ − 7.42 × 10^−2^ ε^2^ + 0.6982 ε − 0.7198	0.99
NC-2wt%HNTs	σ(ε) = 3 × 10^−7^ ε^5^ − 2 × 10^−5^ ε^4^ + 6 × 10^−4^ ε^3^ − 6.3 × 10^−3^ ε^2^ + 0.202 ε + 0.3514	0.98
NC-4wt%HNTs	σ(ε) = 1 × 10^−6^ ε^5^ − 1 × 10^−4^ ε^4^ + 4.57 × 10^−3^ ε^3^ − 9.2 × 10^−2^ ε^2^ + 0.8067 ε − 0.4499	0.98
NC-6wt%HNTs	σ(ε) = 1 × 10^−6^ ε^5^ − 1 × 10^−4^ ε^4^ + 4.2 × 10^−3^ ε^3^ − 7.25 × 10^−2^ ε^2^ + 0.6331 ε − 0.1783	0.98
NC-8wt%HNTs	σ(ε) = 2 × 10^−6^ ε^5^ − 1.735 × 10^−4^ ε^4^ + 6.4 × 10^−3^ ε^3^ − 0.1043 ε^2^ + 0.8216 ε − 0.2194	0.98
NC-10wt%HNTs	σ(ε) = 2 × 10^−6^ ε^5^ − 1.92 × 10^−4^ ε^4^ + 7.5 × 10^−3^ ε^3^ − 0.1213 ε^2^ + 0.8986 ε + 0.0102	0.99

Note: σ and ε denote compressive stress and compressive strain, and σ (ε) refers to compressive stress function in terms of compressive strain.

**Table 8:** Summary of integration results of trendline functions.

Sample	Integration Result of Trendline Function F(x)
PU	σ′(ε) = 1.33 × 10^−7^ ε^6^ − 1.8 × 10^−5^ ε^5^ + 0.975 × 10^−3^ ε^4^ − 2.473 × 10^−2^ ε^3^ + 0.3491 ε^2^ − 0.7198 ε + 0.2781
NC-2wt% HNTs	σ′(ε) = 0.5 × 10^−7^ ε^6^ − 0.4 × 10^−5^ ε^5^ + 1.5 × 10^−4^ ε^4^ − 2.1 × 10^−3^ ε^3^ + 0.101 ε^2^ + 0.3514 ε + 0.1057
NC-4wt% HNTs	σ′(ε) = 0.167 × 10^−6^ ε^6^ − 0.2 × 10^−4^ ε^5^ + 1.1425 × 10^−3^ ε^4^ − 3.067 × 10^−2^ ε^3^ + 4.0335 × 10^−2^ ε^2^ − 0.4499 ε + 0.1253
NC-6wt% HNTs	σ′(ε) = 0.167 × 10^−6^ ε^6^ − 0.2 × 10^−4^ ε^5^ + 1.04 × 10^−3^ ε^4^ − 2.4167 × 10^−2^ ε^3^ + 0.3165 ε^2^ − 0.1783 ε + 0.0192
NC-8wt% HNTs	σ′(ε) = 0.33 × 10^−6^ ε^6^ − 0.347 × 10^−4^ ε^5^ + 1.6 × 10^−3^ ε^4^ − 0.035 ε^3^ + 0.4108 ε^2^ − 0.2194 ε + 0.0221
NC-10wt% HNTs	σ′(ε) = 0.33 × 10^−6^ ε^6^ − 0.384 × 10^−4^ ε^5^ + 1.875 × 10^−3^ ε^4^ − 0.0404 × 10^−2^ ε^3^ + 0.4493 ε^2^ + 0.0102 ε + 0.1018

Note: σ and ε denote compressive stress and compressive strain, and σ′(ε) refers to their corresponding derivative function.

**Table 9:** Density of nanocomposite RE structures.

Sample	Density of PU *ρ_s_* (kg/m^3^)	Density of RE Structure *ρ* (kg/m^3^)
PU	1200	467.49
NC-2	1205	469.43
NC-4	1210	471.38
NC-6	1215	473.33
NC-8	1218	474.50
NC-10	1222	476.06

**Table 10:** Compressive properties of nanocomposite RE structures.

HNT Addition (wt%)	Compressive Strength (MPa)	Compressive Modulus (GPa)	SEA (kJ/kg)
0	3.88 ± 0.036	0.28 ± 0.011	0.44 ± 0.026
2	4.11 ± 0.090	0.35 ± 0.022	0.57 ± 0.015
4	4.96 ± 0.029	0.50 ± 0.010	0.64 ± 0.006
6	5.14 ± 0.035	0.54 ± 0.010	0.65 ± 0.012
8	5.18 ± 0.009	0.61 ± 0.014	0.70 ± 0.007
10	5.20 ± 0.106	0.63 ± 0.013	0.76 ± 0.034

**Table 11:** Shape memory properties of nanocomposite RE structures.

HNT Addition (wt%)	Shape Fixity Ratio *R_f_* (%)	Shape Recovery Ratio *R_r_* (%)	Recovery Time (min)
0	80.21	99.09	3.45
2	83.38	98.66	4.04
4	85.19	97.23	4.29
6	86.96	95.58	4.66
8	88.97	93.42	4.87
10	89.50	90.27	5.02

## Data Availability

The data presented in this study are available on request.

## Supplementary Materials

The following supporting information can be downloaded at: https://www.mdpi.com/article/10.3390/nano14161373/s1, Figure S1: Three-point bending test: (**a**) initial condition, (**b**) after bending; Figure S2: Effect of HNT addition on (**a**) compressive strength along with compressive modulus, as well as (**b**) SEA of PU/HNT nanocomposites; Figure S3: Effects of HNT content on (**a**) shape fixity along with shape recovery ratios and (**b**) recovery time for RE structures
