# Supplementary material for: Mechanical and Shape Memory Properties of Additively Manufactured Polyurethane (PU)/Halloysite Nanotube (HNT) Nanocomposites
Source: Nanomaterials (Basel). 2024 Aug 22;14(16):1373. doi: 10.3390/nano14161373 (PMC11357460; doi:10.3390/nano14161373)
Supplement: Supplementary file 1 [file nanomaterials-14-01373-s001.zip › nanomaterials-3130693-supplementary.pdf]

Supplementary Data

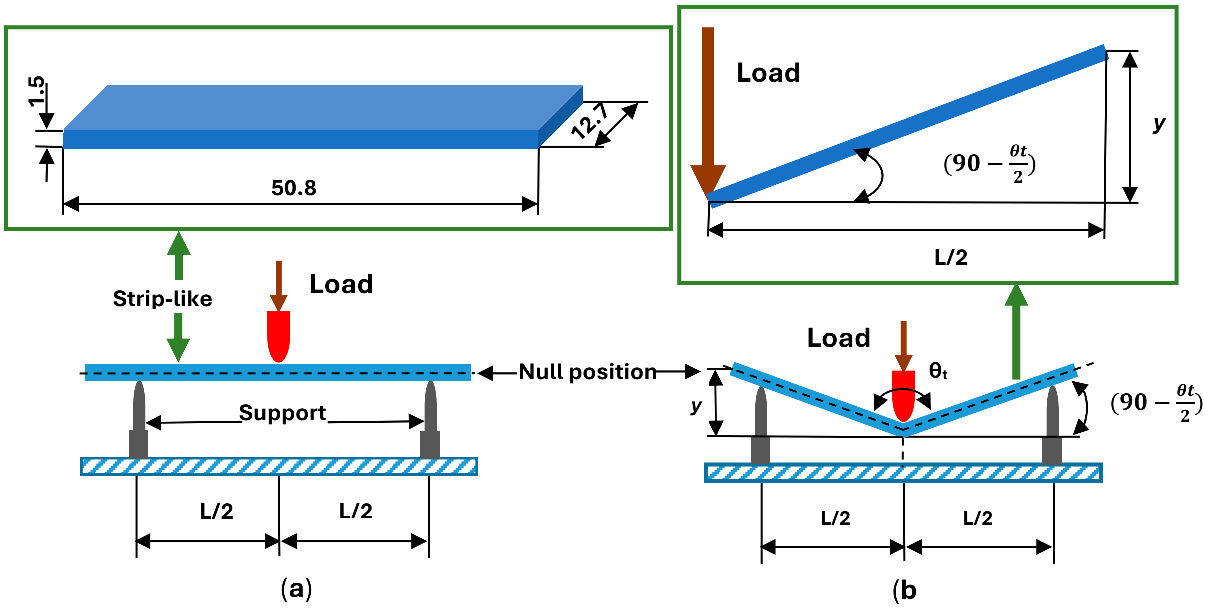

Figure S1. Three-point bending test: (**a**) initial condition, (**b**) after bending.

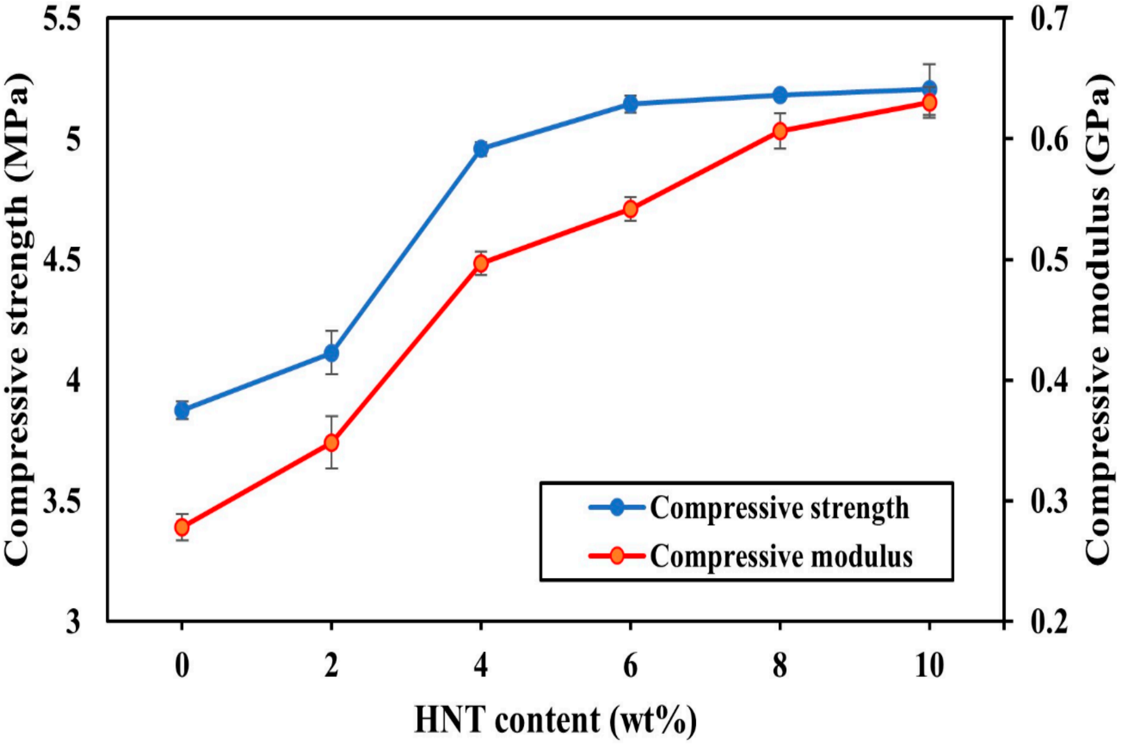

(**a**)

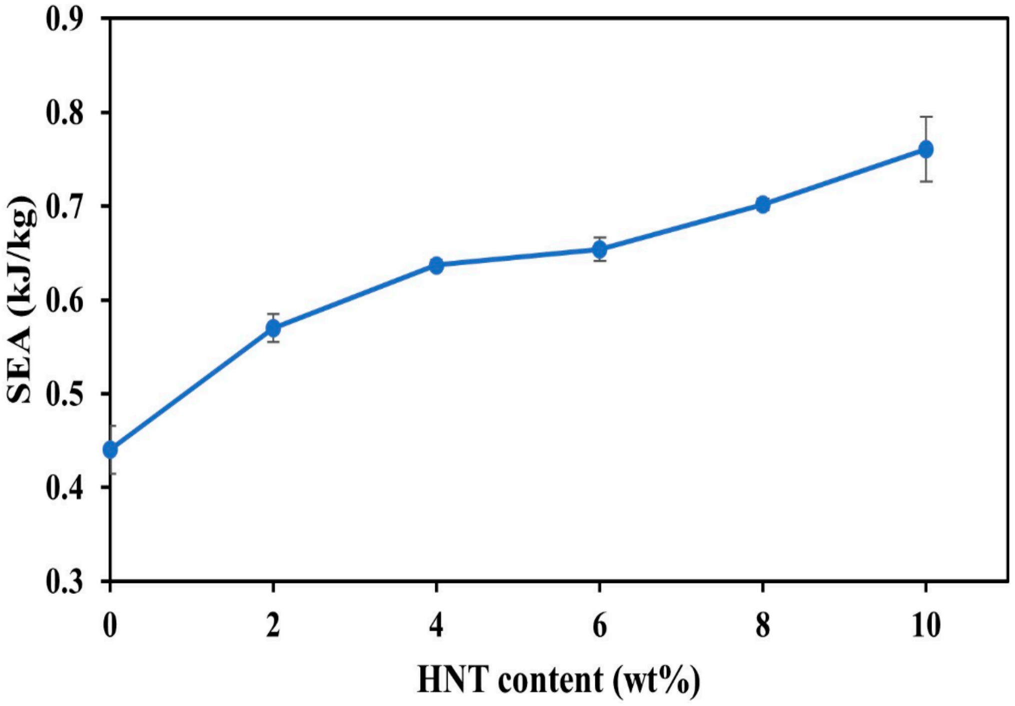

(**b**)

Figure S2. Effect of HNT addition on (**a**) compressive strength along with compressive modulus, as well as (**b**) SEA of PU/HNT nanocomposites.

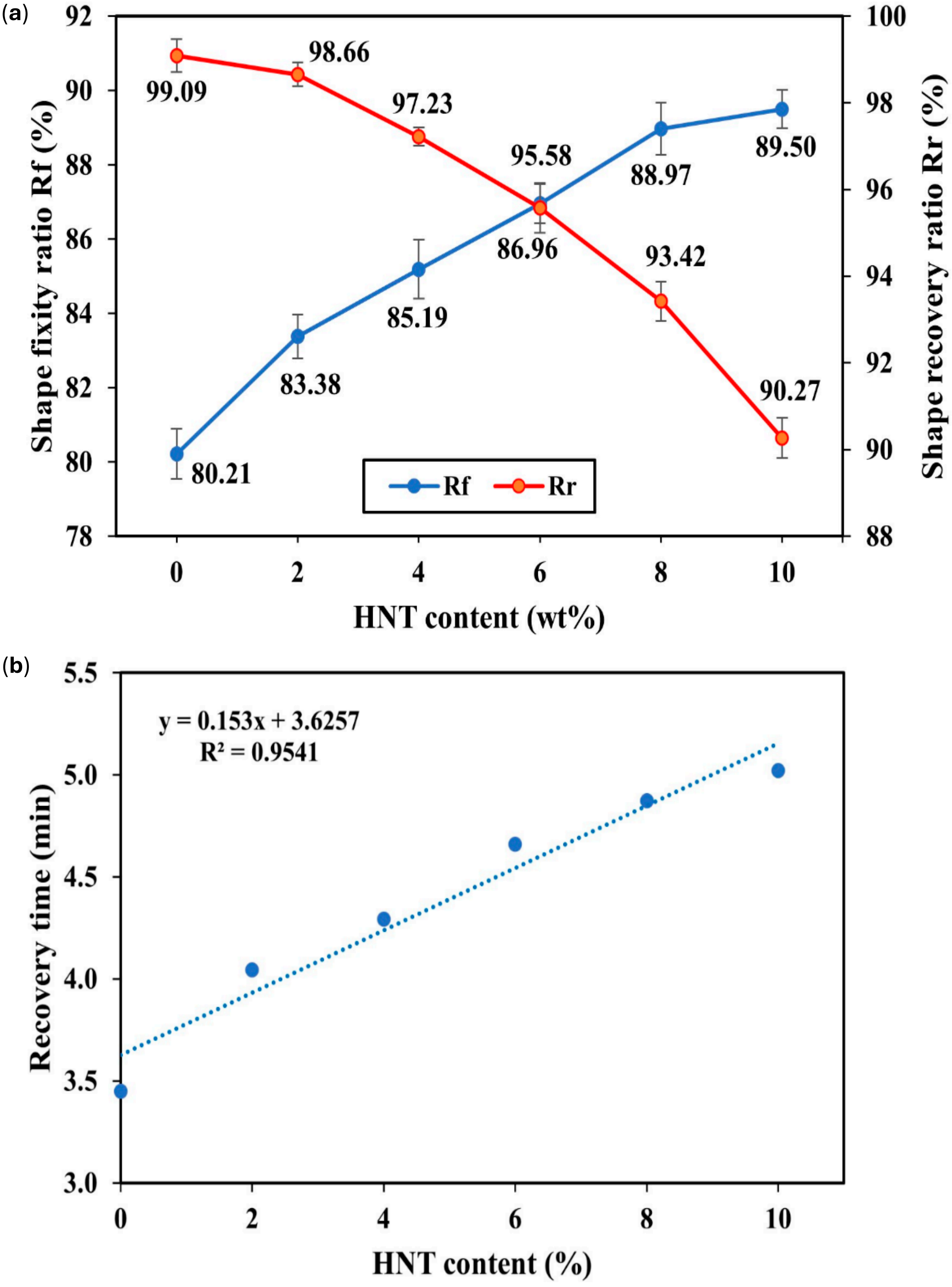

Figure S3. Effects of HNT content on **(a)** shape fixity along with shape recovery ratios and **(b)** recovery time for RE structures.
